# Adenosine A1 receptor activation increases myocardial protein *S*-nitrosothiols and elicits protection from ischemia-reperfusion injury in male and female hearts

**DOI:** 10.1371/journal.pone.0177315

**Published:** 2017-05-11

**Authors:** Qin Shao, Kevin M. Casin, Nathan Mackowski, Elizabeth Murphy, Charles Steenbergen, Mark J. Kohr

**Affiliations:** 1 Department of Cardiology, Renji Hospital, Shanghai Jiaotong University School of Medicine, Shanghai, China; 2 Department of Pathology, School of Medicine, Johns Hopkins University, Baltimore, Maryland, United States of America; 3 Department of Environmental Health and Engineering, Bloomberg School of Public Health, Whiting School of Engineering, Johns Hopkins University, Baltimore, Maryland, United States of America; 4 Systems Biology Center, National Heart, Lung and Blood Institute, Bethesda, Maryland, United States of America; Emory University, UNITED STATES

## Abstract

Nitric oxide (NO) plays an important role in cardioprotection, and recent work from our group and others has implicated protein *S*-nitrosylation (SNO) as a critical component of NO-mediated protection in different models, including ischemic pre- and post-conditioning and sex-dependent cardioprotection. However, studies have yet to examine whether protein SNO levels are similarly increased with pharmacologic preconditioning in male and female hearts, and whether an increase in protein SNO levels, which is protective in male hearts, is sufficient to increase baseline protection in female hearts. Therefore, we pharmacologically preconditioned male and female hearts with the adenosine A1 receptor agonist N6-cyclohexyl adenosine (CHA). CHA administration prior to ischemia significantly improved functional recovery in both male and female hearts compared to baseline in a Langendorff-perfused heart model of ischemia-reperfusion injury (% of preischemic function ± SE: male baseline: 37.5±3.4% vs. male CHA: 55.3±3.2%; female baseline: 61.4±5.7% vs. female CHA: 76.0±6.2%). In a separate set of hearts, we found that CHA increased p-Akt and p-eNOS levels. We also used SNO-resin-assisted capture with LC-MS/MS to identify SNO proteins in male and female hearts, and determined that CHA perfusion induced a modest increase in protein SNO levels in both male (11.4%) and female (12.3%) hearts compared to baseline. These findings support a potential role for protein SNO in a model of pharmacologic preconditioning, and provide evidence to suggest that a modest increase in protein SNO levels is sufficient to protect both male and female hearts from ischemic injury. In addition, a number of the SNO proteins identified with CHA treatment were also observed with other forms of cardioprotective stimuli in prior studies, further supporting a role for protein SNO in cardioprotection.

## Introduction

Protein *S*-nitrosylation (SNO) is a reversible, NO-dependent thiol modification that has been implicated in the regulation of protein function and stability, protein-protein interaction, and cellular localization. Recent work from our group and others has shown that protein SNO increases in male hearts with many different forms of cardioprotective stimuli, including ischemic pre- and post-conditioning [1–10]. The moderate and transient increase in protein SNO observed with acute cardioprotection is thought to contribute to the maintenance of nitroso-redox balance following ischemia-reperfusion (I/R) injury [1–10], in part by shielding critical cysteines from irreversible oxidation. Importantly, we and others have also shown that the protective effects of protein SNO can be blocked with the SNO-specific reducing agent ascorbate [11], but not with soluble guanylate cyclase or protein kinase G inhibition [4, 12–14], supporting a protective role for protein SNO in the setting of I/R injury.

The female heart also exhibits intrinsic protective mechanisms that reduce susceptibility to myocardial damage following I/R injury [15]. Improved post-ischemic functional recovery and reduced infarct size are typically observed in female hearts following I/R injury compared to males [16–19], although this male-female difference has not been observed in all reports [20, 21]. Studies from our group and others support a protective role for NO in the female heart [22–24], and we recently identified a sex difference in myocardial SNO signaling that may be related to sex-dependent cardioprotection [25]. Specifically female hearts exhibited 65% more total SNO proteins, and higher eNOS expression, phosphorylation, and NO production at baseline compared to male hearts. We also found considerable overlap amongst the SNO proteins modified at baseline in female hearts and those modified with cardioprotective stimuli in male hearts. A high number of SNO modified proteins were found in the mitochondria of female hearts at baseline, which exhibited 30% more mitochondrial SNO protein identifications compared to male hearts. A recent study also demonstrated enhanced mitochondrial protein SNO in non-failing human female hearts compared to males [26], suggesting relevance to human physiology.

Although studies from our group and others have demonstrated a protective role for protein SNO in ischemic pre- and post-conditioning and sex-dependent cardioprotection, studies have not examined a role for protein SNO in models of pharmacologic preconditioning in both male and female hearts. Studies also have yet to determine whether an increase in protein SNO levels, which is protective in male hearts, is similarly protective in female hearts and sufficient to further reduce I/R injury beyond the intrinsic protection observed at baseline. Therefore, the goal of this study was to determine whether pharmacologic preconditioning induces cardioprotection from I/R injury by increasing protein SNO levels in male and female hearts. We conducted experiments to determine if a moderate increase in protein SNO affords additional protection in female hearts using an established model of pharmacological preconditioning with the adenosine A1 receptor agonist N6-cyclohexyl adenosine (CHA) [27]. Adenosine A1 receptor stimulation leads to activation of the phosphoinositide 3-kinase/protein kinase B (Akt)/endothelial nitric oxide synthase (eNOS) signaling cascade [28, 29], which we surmised may increase SNO protein levels. Indeed, perfusion with CHA increased phospho-Akt and phospho-eNOS levels, enhanced protein SNO levels and improved functional recovery in both male and female hearts.

## Materials and methods

### Animals

Male and female C57BL/6J mice were obtained from the Jackson Laboratory (Bar Harbor, ME). All animals utilized in this study were between the ages of 12–16 weeks. Mice were housed in a vivarium facility at Johns Hopkins University under specific pathogen-free barrier conditions in rooms that maintain constant temperature, humidity, and a 12-hour light/dark cycle. Animals were provided water and chow *ad libitum*. Each individual cage was supplied with HEPA filtered air and sterile water, and bedding was changed 2–3 times per week. A total of 52 mice were used in this study. For all procedures, mice were anesthetized with a mixture of ketamine (Hofspira, Inc., Lake Forest, IL; 90 mg/kg) and xylazine (Sigma, St. Louis, MO; 10 mg/kg) via intraperitoneal injection, and anticoagulated with heparin (Fresenvis Kabi USA, Lake Zurich, IL). After verifying adequate anesthesia via toe pinch, mice were subsequently euthanized via myocardial excision and exsanguination. This investigation conforms to the *Guide for the Care and Use of Laboratory Animals* published by the United States National Institutes of Health (NIH publication No. 85–23, revised 2011) and was approved by the Institutional Animal Care and Use Committee of Johns Hopkins University.

### Solutions and drugs

Krebs-Henseleit buffer (KHB) consisted of (in mmol/L): NaCl (120), KCl (4.7), KH_2_PO_4_ (1.2), NaHCO_3_ (25), MgSO_4_ (1.2), D-Glucose (11), CaCl_2_ (1.75); pH 7.4. KHB was bubbled with 95%O_2_/5%CO_2_. CHA (Sigma) was used as an adenosine A1 receptor agonist. Ascorbate (Sigma) was used as a SNO-specific reducing agent. All solutions were made fresh on the day of experimentation.

### I/R treatment protocol

**Fig 1:**
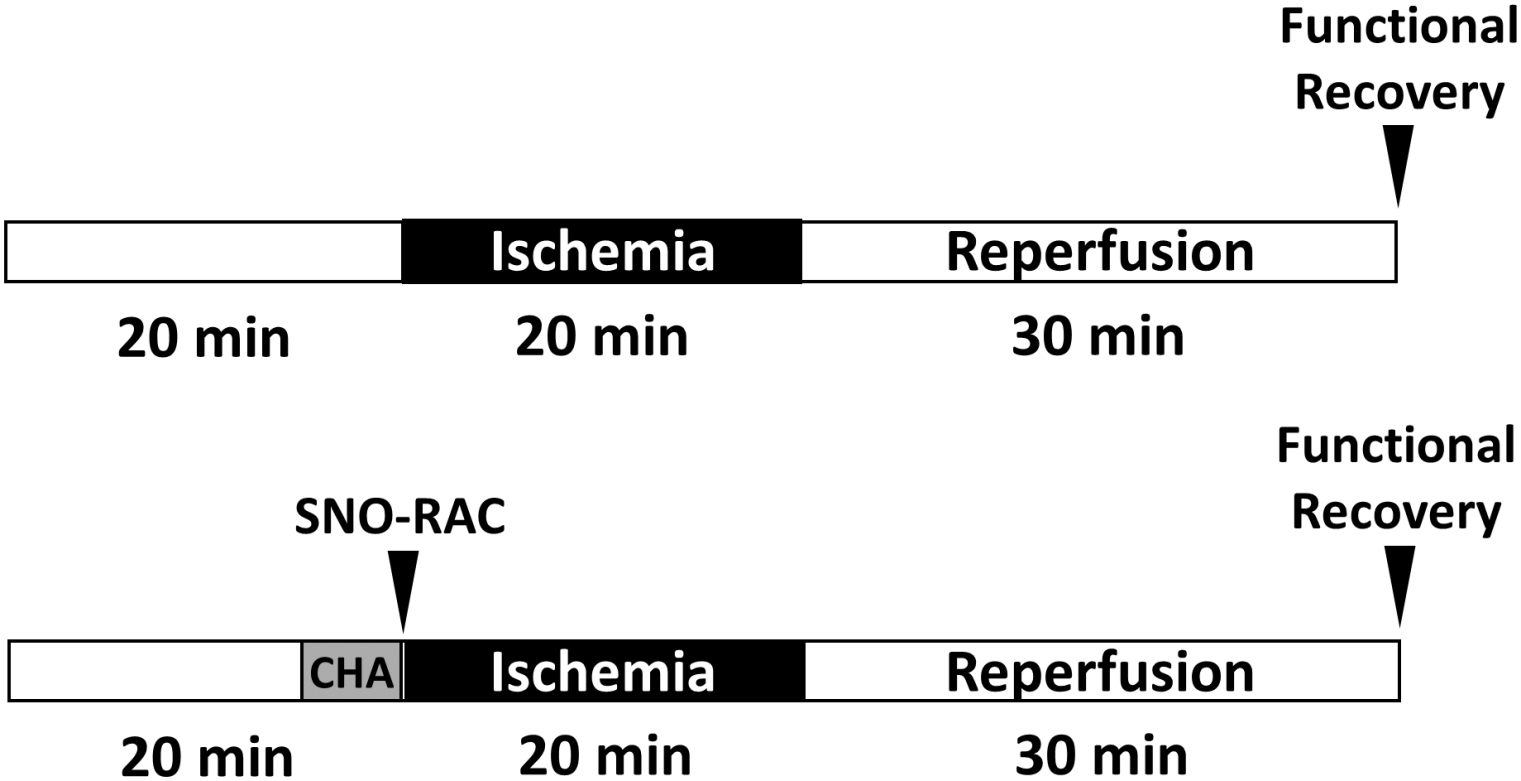
Perfusion protocol for CHA-induced cardioprotection. Hearts were Langendorff-perfused during a 20 minute equilibration period with or without CHA, and then subjected to a 20 minute period of ischemia and 30 minutes of reperfusion.

Hearts were excised from male and female mice and placed in ice-cold KHB. The aorta was cannulated and the heart was perfused in a retrograde fashion on a Langendorff apparatus with KHB at a constant pressure of 100 cm of water at 37°C in the dark, in order to prevent light-induced cleavage of SNO. Male and female hearts were then randomly subjected to an I/R protocol (Fig 1a; 20 minute equilibration period, 20 minute ischemic period, 30 minute reperfusion period) or a CHA-I/R protocol (Fig 1a; 15 min equilibration period, 5 minute CHA perfusion period, 20 minute ischemic period, 30 minute reperfusion period). A latex balloon connected to a pressure transducer was inserted into the left ventricle to measure left ventricular developed pressure (LVDP); LVDP and heart rate were recorded and digitized through a PowerLab system (AD Instruments, Dunedin, New Zealand). The rate pressure product was calculated and used as a measure of cardiac contractile function. Postischemic functional recovery was expressed as a percentage of the preischemic rate pressure product.

### Whole heart homogenate preparation

Whole heart homogenates were prepared as described previously [3, 30]. Briefly, control male and female hearts were excised and rinsed in ice-cold KHB in the dark and snap frozen in liquid nitrogen. A separate set of male and female hearts were Langendorff-perfused with CHA for 5 minutes in the dark and snap frozen in liquid nitrogen. All subsequent procedures were performed in the dark. Hearts were powdered on liquid nitrogen with a mortar and pestle, and resuspended in 1.0 mL of homogenization buffer containing (in mmol/L): sucrose (300), HEPES-NaOH pH 8.0 (250), EDTA (1), neocuproine (0.1), and Triton-X 100 (0.5%). An EDTA-free protease inhibitor tablet (Roche, Indianapolis, IN) was added just before use. Samples were then homogenized using a Dounce glass homogenizer on ice and centrifuged at 14,000 g for 30 minutes to pellet debris. The supernatant was recovered as total crude homogenate. Protein concentration was determined using the Bradford protein assay. Total homogenates were then aliquoted and stored at -80°C.

### SNO-RAC

SNO sites were identified using the SNO-RAC protocol, as previously described [3, 30]. Briefly, samples (whole heart homogenate; 1 mg) were diluted in HEN buffer containing (in mmol/L): **H**EPES-NaOH pH 8.0 (250), **E**DTA (1), and **N**eocuproine (0.1) with 2.5% SDS and an EDTA free protease inhibitor tablet. All buffers were degassed before use to prevent oxidation of the resin; subsequent procedures were also performed in the dark. Homogenates were incubated with 50 mmol/L N-ethylmaleimide for 20 minutes at 56°C to block unmodified thiol groups from modification; iodoacetamide was removed via acetone precipitation. Samples were then resuspended in HEN with 1% SDS (HENS). Thiopropyl sepharose resin (GE Healthcare, Piscataway, NJ) was rehydrated for 25 minutes in HPLC-grade water. Following rehydration, 25 μL of the resin slurry was added to a Handee Mini Spin Column (Thermo Fisher, Waltham, MA) and washed with 5 x 0.5 mL HPLC-grade water, followed by 10 x 0.5 mL HEN buffer. Blocked samples were then added to the thiopropyl sepharose-containing spin column along with 20 mmol/L ascorbate, and rotated for 4 hours in the dark at room temperature. Resin-bound proteins were washed with 8 x 0.5 mL HENS buffer, followed by 4 x 0.5 mL HENS buffer diluted 1:10. Samples were then subjected to trypsin digestion (sequencing grade modified; Promega, Madison, WI) overnight at 37°C with rotation in 50 mmol/L NH_4_HCO_3_. Resin-bound peptides were then washed with 5 x 0.5 mL HENS buffer 1:10, 5 x 0.5 mL NaCl, 5 x 0.5 mL 80% acetonitrile, and 5 x 0.5 mL HPLC-grade water. Resin-bound peptides were then eluted for 15 minutes at room temperature in elution buffer containing (in mmol/l): dithiothreitol (20) and NH_4_CO_3_ (10). The resin was then washed with an additional volume of elution buffer. All fractions were combined and concentrated by SpeedVac (Thermo Fisher), resuspended in 50 mmol/L NH_4_HCO_3_, and all detergent was removed using HiPPR columns (Thermo Fisher) per the manufacturer’s instruction. Samples were then resuspended in 0.1% formic acid and cleaned with C_18_ column ZipTips (EMD Millipore, Billerica, MA) prior to liquid chromatography-tandem mass spectrometry (LC-MS/MS) analysis.

### LC-MS/MS analysis and database search

LC-MS/MS was performed using an Eksigent nanoLC-Ultra 1D plus system (Dublin, CA) coupled to an LTQ Orbitrap Elite mass spectrometer (Thermo Fisher) using CID fragmentation. Peptides were first loaded onto a Zorbax 300SB-C_18_ trap column (Agilent, Santa Clara, CA) at a flow rate of 6 μL/minute for 6 minutes, and then separated on a reversed-phase PicoFrit analytical column (New Objective, Woburn, MA) using a 40-minute linear gradient of 5–40% acetonitrile in 0.1% formic acid at a flow rate of 250 nL/minute. LTQ Orbitrap Elite settings were as follows: spray voltage 1.5 kV, and full MS mass range: m/z 230 to 2000. The LTQ Orbitrap Elite was operated in a data-dependent mode (i.e., one MS1 high resolution [60,000] scan for precursor ions followed by six data-dependent MS/MS scans for precursor ions above a threshold ion count of 2000 with collision energy of 35%). Raw files generated from the LTQ Orbitrap Elite were analyzed using Proteome Discoverer 1.4 (Thermo Fisher) with the MASCOT database search engine. The following search criteria were used: database, Swiss-Prot (Swiss Institute of Bioinformatics); taxonomy, Mus musculus (mouse); enzyme, trypsin; miscleavages, 3; variable modifications, Oxidation (M), Nethylmaleimide (C), Deamidation (NQ); MS peptide tolerance, 25 ppm; MS/MS tolerance, 0.8 Da. Peptides were filtered at a false discovery rate (FDR) of 1%.

### Label-free peptide quantification

The label-free peptide quantification feature of Proteome Discoverer 1.4 was used to determine the ratio or relative abundance for a given SNO-modified residue. This label-free peptide quantification function uses a proprietary algorithm to calculate the area for each peptide based upon the area-under-the-curve peak for a given peptide from each LC-MS/MS run. Quantitative ratios were then obtained by normalizing the peptide peak areas against a chosen reference (i.e., baseline male heart for common peptides). The resulting ratios reflect the relative quantity of a peptide (and hence the corresponding SNO level) in different samples.

### Western blot

Samples were separated on a 4–12% Bis-Tris SDS-PAGE gel and transferred onto a nitrocellulose membrane (Life Technologies). Membranes were blocked with 5% (w/v) nonfat dried milk in Tris-buffered saline with 0.1% Tween-20 for one hour, and subsequently incubated with primary antibodies against phospho-Akt Ser473 (1:1000, Cell Signaling Technology, Danvers, MA, 4060S), total Akt (1:1000, Cell Signaling Technologies, 4691S), phospho-eNOS Ser1177 (1:500; Cell Signaling), total eNOS (1:250; Santa Cruz Biotechnology, Dallas, TX), or GSNOR (1:1000; Santa Cruz Biotechnology). Membranes were then probed with the corresponding secondary antibodies for 1 hour and visualized by electrogenerated chemiluminescence (Life Technologies). Densitometry was assessed using ImageJ software (National Institutes of Health, Bethesda, MD).

### GSNO-R activity assay

GSNO-R activity was assessed in whole heart homogenates as previously described [31, 32]. Briefly, male and female hearts were rinsed in KHB and snap frozen in liquid nitrogen. Hearts were then homogenized in cell lysis buffer (Cell Signaling, Danvers, MA) with protease/inhibitor cocktail (Cell Signaling) using a Polytron (Kinematica Inc.). Homogenates (100 μg) were then diluted in assay buffer (in mmol/L): Tris-HCl pH 8.0 (20), EDTA (0.5), neocuproine (0.5) with 0.1% NP-40 and protease/phosphatase inhibitor cocktail (Cell Signaling). NADH (200 μmol/L) and GSNO (400 μmol/L) were then added to initiate the reaction, and NADH consumption was monitored via absorbance at 340 nm over 30 minutes at 25°C. GSNO-R activity was then calculated as the rate of NADH consumption in samples containing GSNO, after subtracting the background rate of NADH consumption in samples that did not contain GSNO.

### Amplex Red assay

H_2_O_2_ production was assessed in post-ischemic male and female hearts using the Amplex Red H_2_O_2_ production assay kit (Thermo Fisher) per the manufacturer’s instruction. Briefly, samples (whole heart homogenate; 100 μg) were diluted in Amplex Red reaction buffer. The reaction was then initiated with the addition of horseradish peroxidase and Amplex Red reagent, and H_2_O_2_ production was followed for 30 minutes at 25°C. The slope of the line over the 30 minute incubation period was used to calculate the rate of H_2_O_2_ production. A standard H_2_O_2_ curve was used to determine the concentration of H_2_O_2_ production. To assess H_2_O_2_ production with purified alpha-ketoglutarate dehydrogenase (alpha-KGDH), the same protocol was used with the purified enzyme complex (Sigma Aldrich).

### Statistics

Results are expressed as the mean±SEM. Statistical significance (p<0.05) was determined between groups using a Student’s *t*-test for two groups or a two-way ANOVA with Tukey’s multiple comparison correction for multiple groups.

## Results

### CHA improves post-ischemic functional recovery in male and female hearts

**Fig 2:**
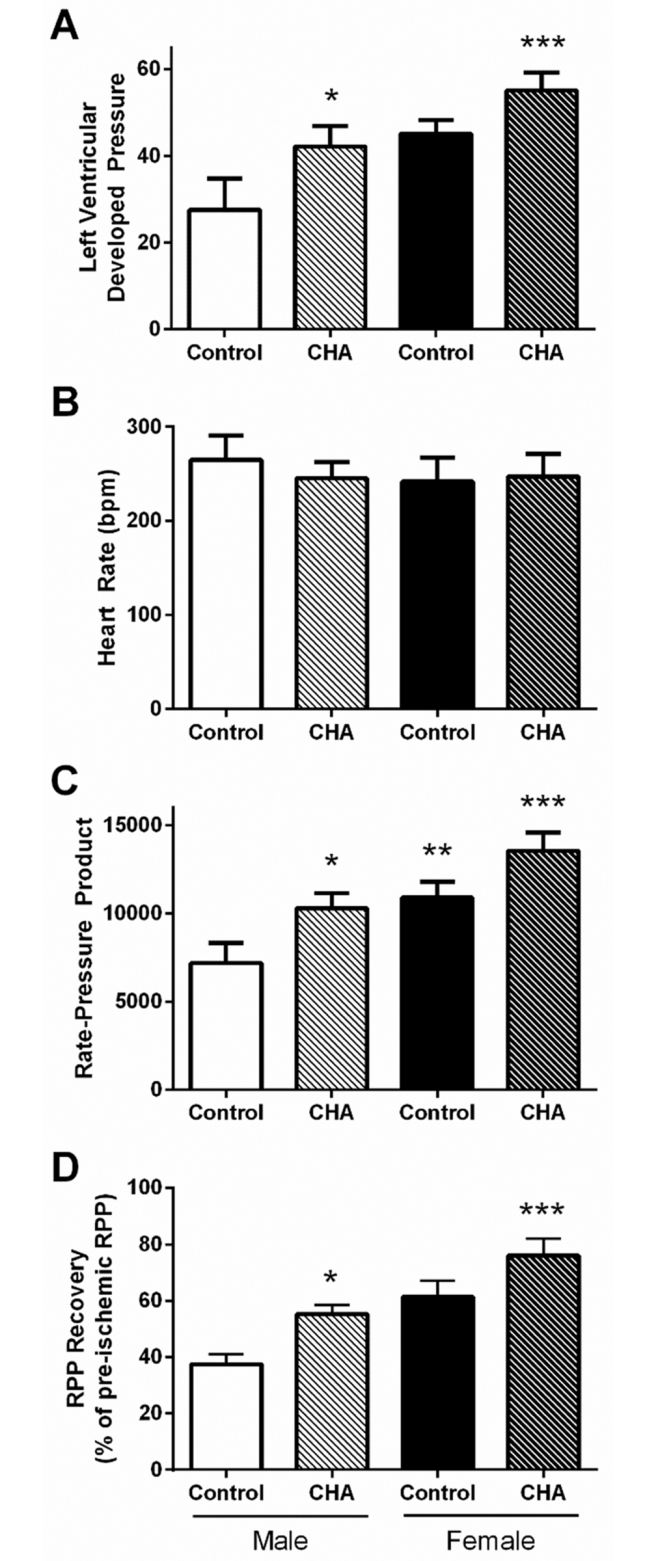
CHA increases post-ischemic functional recovery in male and female hearts. (A) Left ventricular developed pressure (LVDP), (B) heart rate, (C) rate-pressure product (RPP), and (D) functional recovery in Langendorff-perfused control and CHA-perfused male and female hearts following 20 minutes of ischemia and 30 minutes of reperfusion (male control: clear bar; male CHA: clear hashed bar; female control: black bar; female CHA: black hashed bar; n = 3 hearts/group; *p<0.05 vs. male control; **p<0.05 vs. male control, female CHA; ***p<0.05 vs. male control, male CHA, female control).

Male and female hearts were subjected to I/R injury via Langendorff perfusion with or without the adenosine A1 receptor agonist CHA (Fig 1), and post-ischemic functional recovery was assessed after 30 minutes of reperfusion. Measurement of initial hemodynamic parameters in male and female hearts perfused with or without CHA revealed no difference in baseline LVDP, heart rate, or rate-pressure product (Table 1). Following 20 minutes of ischemia and 30 minutes of reperfusion, contractile function was significantly impaired in both male and female hearts (Fig 2), and consistent with our previous study [25], control female hearts exhibited improved post-ischemic functional recovery compared to control male hearts. Perfusion with CHA for 5 minutes prior to the onset of ischemia significantly improved post-ischemic functional recovery in both male and female hearts (Fig 2), with CHA-perfused female hearts exhibiting the highest recovery of function.

**Table 1:** Baseline contractile parameters with and without CHA.

	*Treatment*	*LVDP*	*Heart Rate*	*Rate-Pressure Product*
***Male***	*Control*	76.7±1.3	250.7±10.6	19195±499.0
	*CHA*	77.8±3.0	230.0±3.4	17911±865.0
***Female***	*Control*	69.0±4.2	271.9±9.1	18789±1510
	*CHA*	69.1±3.0	268.1±13.6	18103±1592

Baseline left ventricular developed pressure, heart rate and rate-pressure product in male and female hearts perfused with and without CHA.

### CHA perfusion increases phospho-Akt and phospho-eNOS levels in male and female hearts

**Fig 3:**
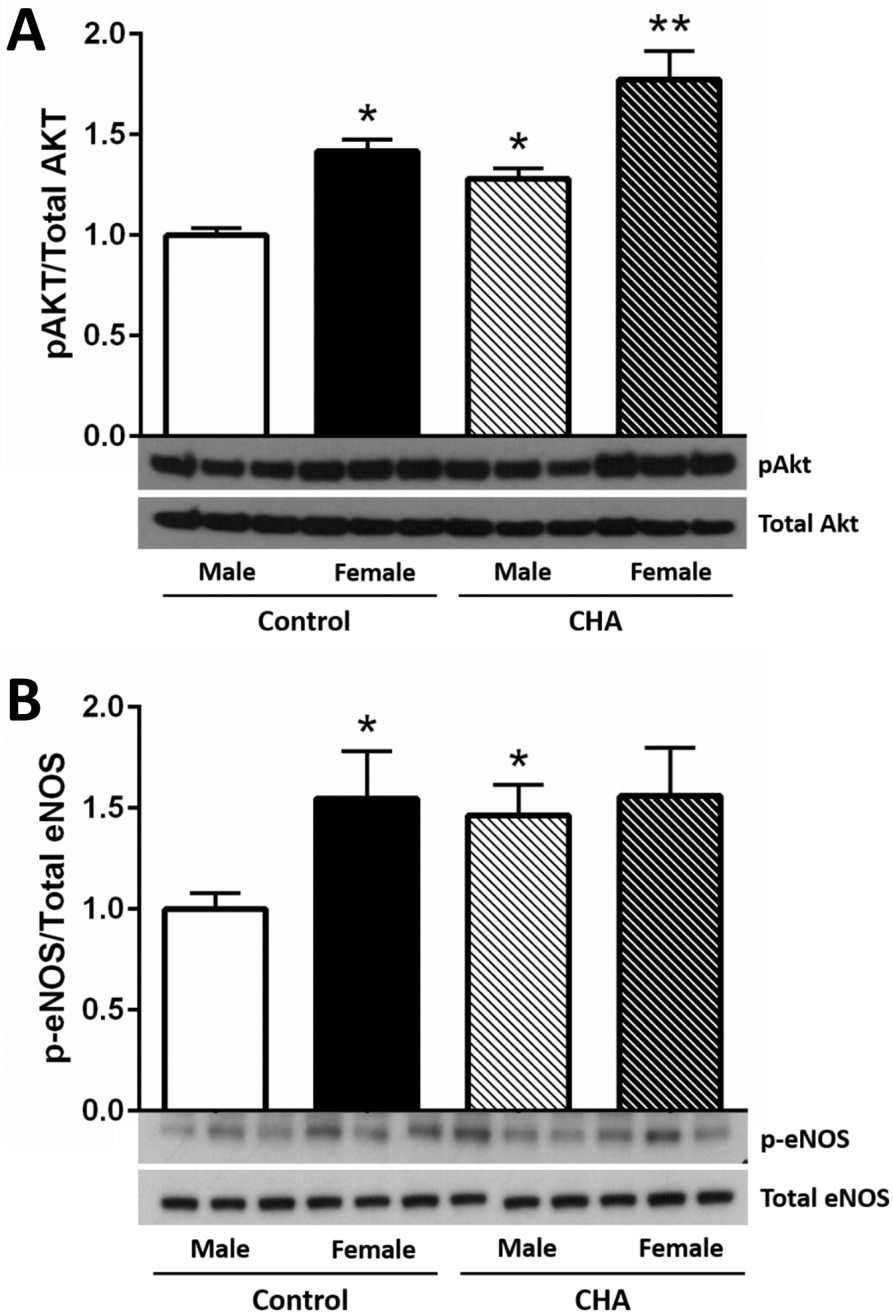
CHA increases phosphorylated Akt and eNOS levels in male and female hearts. (A) Western blot depicting phosphorylated (Ser473) and total Akt levels from control and CHA-perfused male and female hearts (male control: clear bar, female control: black bar, male CHA: clear hashed bar, female CHA: black hashed bar; n = 6 hearts/group; *p<0.05 vs. male control, female CHA; **p<0.05 vs. male control, male CHA, female control). (B) Western blot depicting phosphorylated (Ser1177) and total eNOS levels from control and CHA-perfused male and female hearts (male control: clear bar, female control: black bar, male CHA: clear hashed bar, female CHA: black hashed bar; n = 6 hearts/group; *p<0.05 vs. male control, female CHA).

Adenosine A1 receptor stimulation has been shown to activate the Akt signaling cascade [28, 29]. Therefore, we next examined Akt phosphorylation at Ser473. Consistent with previous studies [33], control female hearts exhibited higher phospho-Akt levels compared to control male hearts, independent of total Akt levels (Fig 3a). Perfusion with CHA for five minutes significantly increased phospho-Akt levels in both male and female hearts, with CHA-perfused female hearts exhibiting the highest phospho-Akt levels (Fig 3a). Consistent with the CHA-induced increase in phospho-Akt levels, we also found that perfusion with CHA significantly increased eNOS phosphorylation at Ser1177 in male hearts (Fig 3b). These findings are in agreement with previous studies demonstrating enhanced phospho-Akt and phospho-eNOS levels with adenosine [28, 29]. However, phospho-eNOS levels did not change with CHA in female hearts (Fig 3b), but phospho-eNOS levels were already higher in control and CHA-perfused female hearts compared to control male hearts. These results support the potential for an alternative mechanism of protection in female hearts.

### CHA perfusion enhances protein SNO levels in male and female hearts

**Fig 4:**
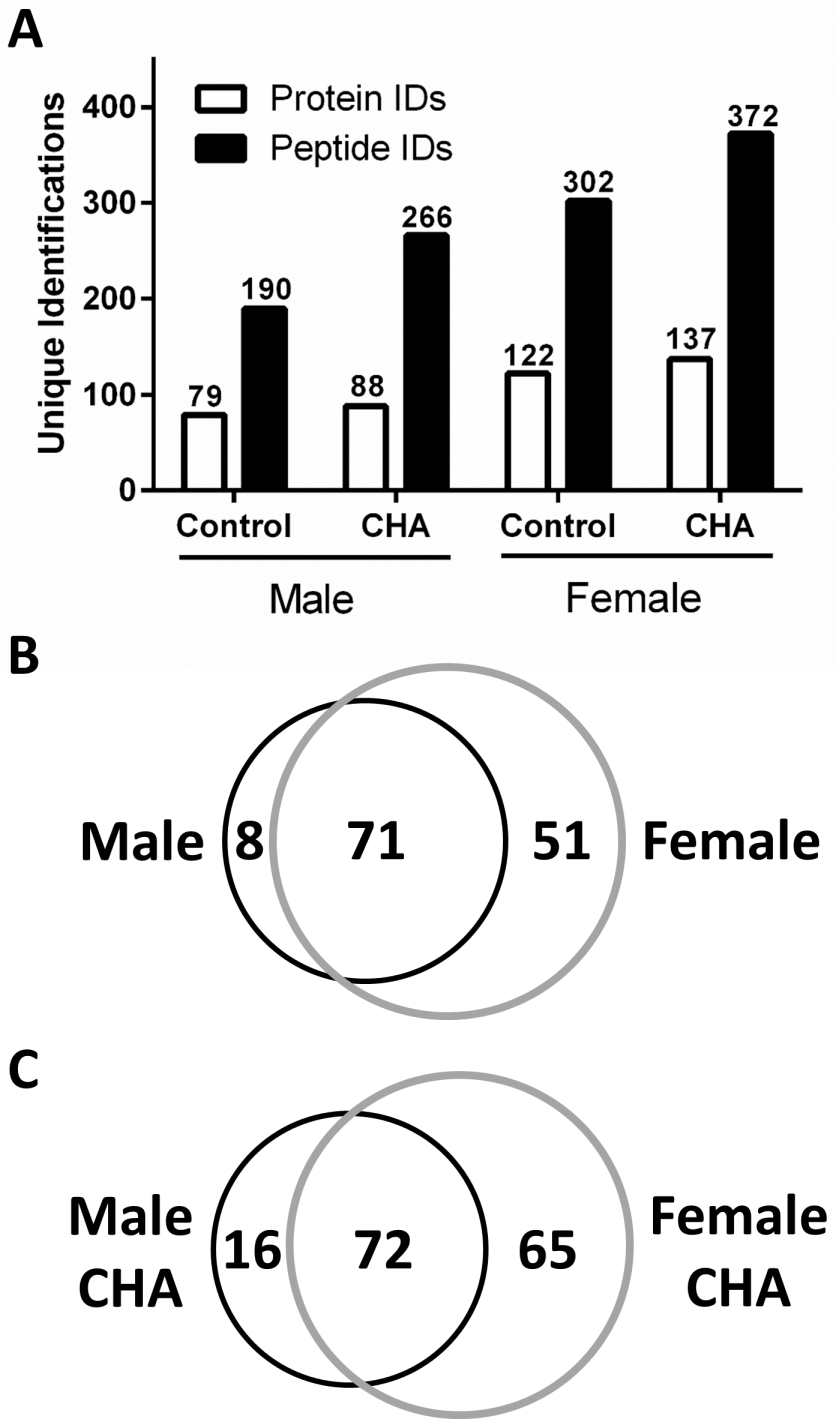
CHA increases SNO protein levels in male and female hearts. (A) Total number of SNO protein and peptide identifications at baseline and with CHA perfusion in male and female hearts as assessed via SNO-RAC in tandem with LC-MS/MS (protein IDs: clear bars; peptide IDs: black bars; n = 7–8 hearts/group; FDR: 1%). (B) Venn diagram depicting common and unique SNO protein identifications at baseline in male (black circle) and female (grey circle) hearts; unique SNO protein identifications were only identified in one group (i.e., male or female). (C) Venn diagram depicting common and unique SNO protein identification in CHA-perfused male (black circle) and female hearts (grey circle).

Since we detected increased phospho-Akt and phospho-eNOS levels with CHA perfusion, we next examined whether this could produce a corresponding increase in protein SNO levels in male and female hearts. We utilized SNO-RAC in tandem with mass spectrometry as a high-throughput approach to identify specific SNO sites in male and female hearts. In total, we identified 130 different SNO proteins in control male and female hearts (S1 and S2 Tables), and consistent with our previous study [25], we found that female hearts exhibited over 50% more unique SNO protein identifications compared to male hearts (Fig 4a). Of the proteins identified in control hearts, 51 were unique to female hearts (i.e., proteins were detected in at least one of eight female samples, but not detected in any male samples) and eight were unique to male hearts (i.e., proteins were detected in at least one of eight male samples, but not detected in any female samples) (Fig 4b). SNO protein identifications that were unique to the baseline female heart included the adenine nucleotide translocase (ANT), ATP synthase ε, dihydrolipoyl dehydrogenase, peroxyredoxin-6, protein DJ-1, and tripartite motif-containing protein 72 (TRIM72).

**Fig 5:**
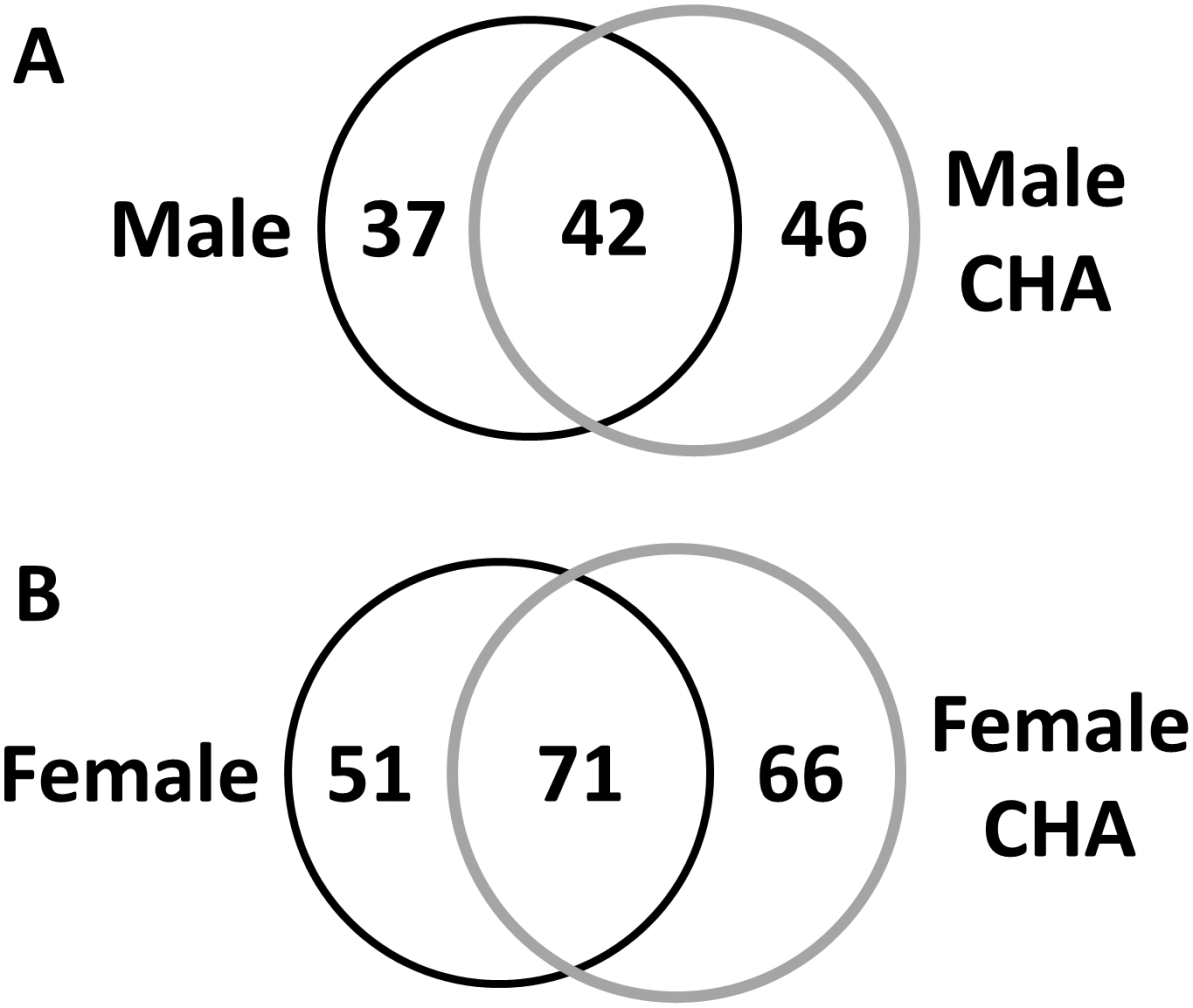
CHA-induced changes in protein SNO in male and female hearts. (A) Venn diagram depicting common and unique SNO protein identifications in male control hearts (black circle) compared to CHA-perfused male hearts (grey circle). (B) Venn diagram depicting common and unique SNO protein identifications in female control hearts (black circle) compared to CHA-perfused female hearts (grey circle).

Following myocardial perfusion with CHA for five minutes, we identified a total of 165 unique SNO proteins in male and female hearts (S3 and S4 Tables), noting a modest increase in protein SNO levels in both male (11.4%) and female (12.3%) hearts compared to control (Fig 4a). Of the proteins identified in CHA-perfused hearts, 65 were unique to female hearts and 16 were unique to male hearts (Fig 4c). A number of SNO proteins that were not detected at baseline in male and female hearts were found to be modified after CHA perfusion in both sexes (Table 2), including cysteine and glycine rich protein 3, cytochrome C oxidase 6B1, reactive species modulator 1, and voltage-dependent anion channel (VDAC) 3. Comparison of control and CHA-perfused male hearts revealed 37 unique SNO proteins in control hearts vs. 46 in CHA-perfused hearts (Fig 5a). Interestingly, a number of the unique SNO proteins identified in control female hearts were identified in CHA-perfused male hearts, including ANT, glutathione S-transferase kappa 1, metallothionein-1 and peroxyredoxin-6. For control and CHA-perfused female hearts, we detected 51 SNO proteins in control hearts versus 66 in CHA-perfused hearts (Fig 5b).

**Table 2:** Unique SNO protein identifications from CHA-perfused hearts.

*Protein Name*	*Protein ID*	*SNO Cysteine*	
		*Male* *CHA*	*Female* *CHA*
40S ribosomal protein S17	P63276	35	35
BAG family molecular chaperone regulator 3	Q9JLV1	185	185
BRI3-binding protein	Q8BXV2	218	218
Cysteine and glycine-rich protein 3	P50462	25, 120	25, 58, 120
Cytochrome c oxidase subunit 6B1	P56391	54	54
Filamin-binding LIM protein 1	Q71FD7	35	36
Fructosamine-3-kinase	Q9ER35	130	130
Laminin subunit beta-2	Q61292	887, 894, 897, 906, 955, 957	906, 955, 957
Laminin subunit gamma-1	P02468	314, 349, 914, 917, 930	314, 349, 914, 917, 930
Membrane-associated progesterone receptor component 2	Q80UU9	75	75
Microsomal glutathione S-transferase 1	Q91VS7	50	50
Myosin light polypeptide 6	Q60605	2	2
Myosin-6	Q02566	37	37, 949, 1342
Nascent polypeptide-associated complex subunit alpha, muscle-specific form	P70670	1763	1763
Perilipin-4	O88492	696	696
Poly(rC)-binding protein 1	P60335	54	54
Protein NDRG2	Q9QYG0	371	371
Reactive oxygen species modulator 1	P60603	15	15
Receptor expression-enhancing protein 5	Q60870	14	14
Sarcospan	Q62147	157	157
Signal peptidase complex subunit	Q9CYN2	26	26
Tubulin beta-5 chain	P99024	303, 354	11, 303, 354
Voltage-dependent anion-selective channel protein 3	Q60931	65	2, 8, 65

Unique SNO protein identifications from CHA-perfused male and female hearts as assessed via SNO-RAC in tandem with LC-MS/MS (n = 7 hearts/group; FDR: 1%); these SNO protein identifications were not observed in control male or control female samples. ‘SNO cysteine’ represents the modified residue.

### SNO protein targets in CHA-induced cardioprotection vs. other forms of cardioprotection

**Fig 6:**
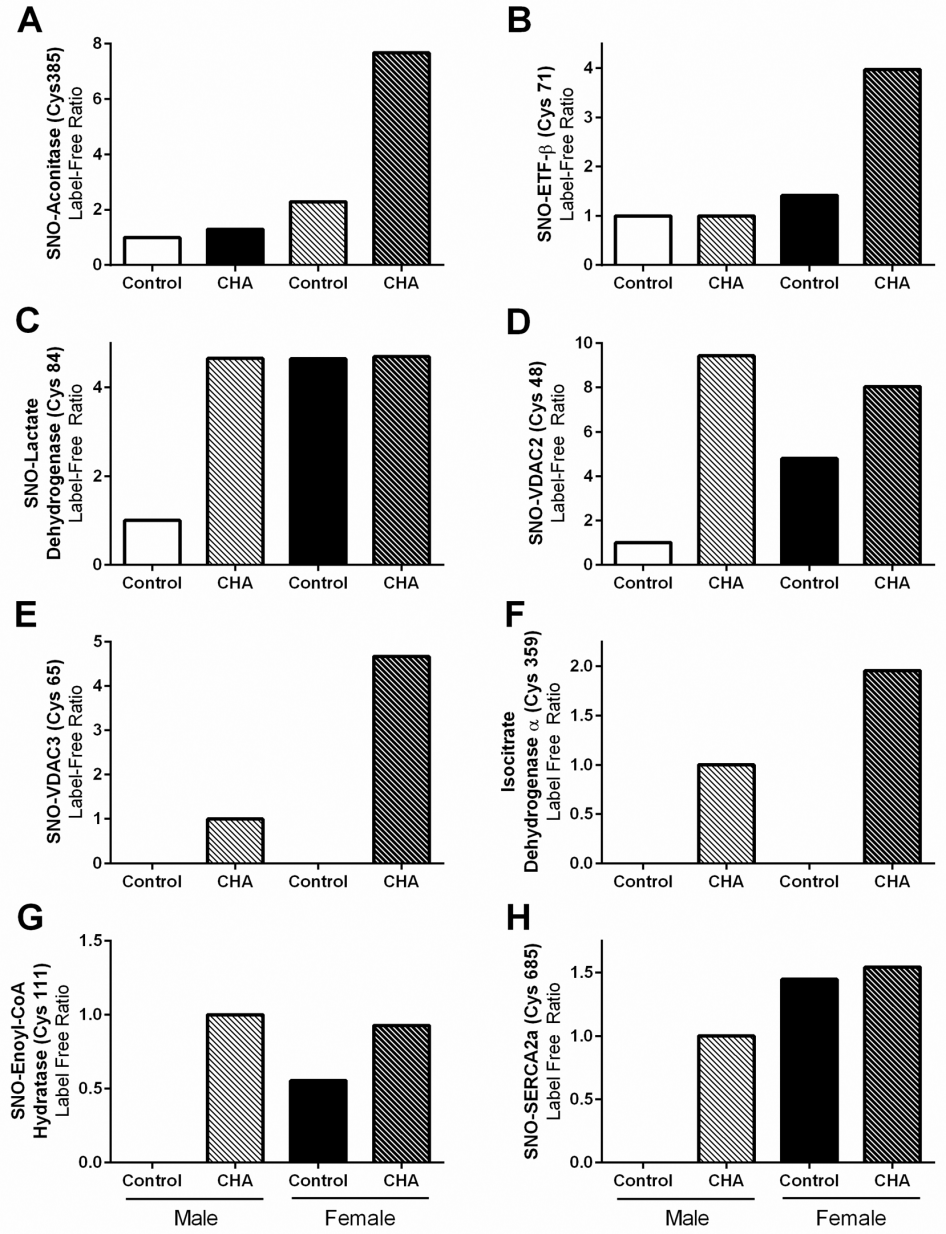
Label-free SNO protein levels for various targets. For common SNO proteins that were identified in male and female hearts, SNO levels were assessed via label-free peptide quantification for (A) aconitase, (B) electron transfer flavoprotein β, (C) lactate dehydrogenase, (D) voltage-dependent anion channel 2, (E) voltage-dependent anion channel 3, (F) isocitrate dehydrogenase α, (G) enoyl-CoA hydratase, and (H) sarcoplasmic reticulum Ca^2+^-ATPase 2a (male control: clear bar, male CHA: clear hashed bar, female control: black bar, female CHA: black hashed bar). Please note that for groups that do not contain a bar, the SNO peptide indicated was not detected.

In previous studies, we noted that protein SNO levels increased with a number of different cardioprotective interventions in the male heart, including ischemic pre- and post-conditioning [1–4]. Therefore, we next compared the SNO protein identifications from male and female hearts perfused with CHA to the SNO proteins that were identified in previous studies also utilizing SNO-RAC with ischemic pre- and post-conditioned male hearts [3, 4]. We found a number of proteins that are consistently SNO modified, many at the same cysteine residue(s), irrespective of the model of cardioprotection (Table 3). SNO targets included cytochrome b-c1 complex subunit 1, L-lactate dehydrogenase, malate dehydrogenase, and triosphosphate isomerase. We also used label-free peptide quantification to quantify the SNO levels of common proteins identified in more than one treatment group, focusing specifically on SNO proteins that were previously identified with other forms of cardioprotection. Interestingly, we found that these SNO proteins clustered into four distinct groups based upon their detection with each of the four experimental treatments. In the first group, protein SNO levels were low at baseline in both male and female hearts, and increased only in the female heart with CHA perfusion. These proteins included aconitase (Fig 6a) and electron transfer flavoprotein beta (Fig 6b). In the second group, SNO levels were low at baseline in male hearts, but were considerably higher in baseline female hearts and in CHA-perfused male and female hearts. Protein targets included L-lactate dehydrogenase (Fig 6c) and VDAC 2 (Fig 6d). The third group included proteins that were only detected in CHA-perfused male and female hearts, and these proteins included VDAC3 (Fig 6e) and isocitrate dehydrogenase α (Fig 6f). Finally, the fourth group contained SNO proteins that were not detected at baseline in the male heart, but were detected in the baseline female heart and in CHA-perfused male and female hearts. These proteins included enoyl-CoA hydratase (Fig 6g) and SERCA2a (Fig 6h).

**Table 3:** Common SNO protein identifications resulting from different cardioprotective interventions.

*Protein Name*	*Protein ID*	*SNO Cysteine*					
		*Male* *Baseline*	*Female* *Baseline*	*Male* *CHA*	*Female* *CHA*	*Male* [3] *IPC*	*Male* [4] *PostC*
Aconitase	Q99KI0	385	385	385	385	385	385
Aspartate aminotransferase, mitochondrial	P05202	295	295	ND	295	295	295
Cytochrome b-c1 complex subunit 1, mitochondrial	Q9CZ13	268, 380	268, 380	268, 380	268, 380	268, 380	380
Electron transfer flavoprotein subunit alpha, mitochondrial	Q99LC5	53, 155	53, 155	ND	155	155	ND
Electron transfer flavoprotein subunit beta, mitochondrial	Q9DCW4	71	71	71	71	66, 71	ND
Enoyl-CoA hydratase, mitochondrial	Q8BH95	225	111, 225	111, 225	111, 225	225	111, 225
Fructose-bisphosphate aldolase A	P05064	339	339	339	339	339	339
Glyceraldehyde-3-phosphate dehydrogenase	P16858	150, 154, 245	150, 154, 245	150, 154, 245	150, 154, 245	150, 154, 245	150, 154, 245
Isocitrate dehydrogenase [NAD] subunit alpha	Q9D6R2	ND	ND	359	359	331	331
Isocitrate dehydrogenase [NADP], mitochondrial	P54071	113, 402	113, 402	ND	113, 402	308, 402	308, 402
L-lactate dehydrogenase A chain	P06151	84, 163	84, 163	84, 163	84, 163	84, 163	163
Malate dehydrogenase, cytoplasmic	P14152	137, 154	137, 154	137	137, 154	137	ND
Malate dehydrogenase, mitochondrial	P08249	89, 93, 275, 285	89, 93, 211, 275, 285	89, 93, 275	89, 93, 211, 275, 285	275	89, 275
Mitochondrial complex I-75 kDa	Q91VD9	75, 92	75, 92	75, 92	92, 726	92	75
Myosin light chain 1	P09542	191	85, 191	ND	85, 191	ND	ND
Propionyl CoA carboxylase alpha chain	Q91ZA3	107	107	107	107	107	107
Sarcoplasmic/endoplasmic reticulum calcium ATPase 2	O55143	349, 364, 447, 471	344, 349, 364, 447, 471, 635, 669	364, 635	344, 364, 447, 635, 998	498	344, 349, 364
Serum albumin	P07724	77, 289, 416, 500, 501	77, 289	77, 289	77, 289	ND	ND
Succinate dehydrogenase [ubiquinone] flavoprotein, mitochondrial	Q8K2B3	536	89, 536, 654	654	536, 654	536	654
Succinyl-CoA ligase alpha	Q9WUM5	172, 181	172, 181	172, 181	172	60	172, 181
Succinyl-CoA ligase subunit beta, mitochondrial	Q9Z2I9	430	152, 158, 270, 430	152, 158, 430	152, 158, 430	430	158, 430
Triosephosphate isomerase	P17751	117, 268	117, 268	117, 268	117, 268	67, 218	117, 268
Voltage-dependent anion-selective channel protein 1	Q60932	245	245	245	245	245	245
Voltage-dependent anion-selective channel protein 2	Q60930	48	48	48	48	48	48

Common SNO protein identifications resulting from different cardioprotective interventions (i.e., ischemic pre- and post-conditioning). SNO proteins were identified in male and female hearts (as noted on the top of the table), using SNO-RAC in tandem with LC-MS/MS. ‘SNO cysteine’ represents the modified residue; ‘ND’ indicates that the protein was not identified in the given sample set.

### S-nitrosoglutathione reductase activity is increased with CHA perfusion in male and female hearts

**Fig 7:**
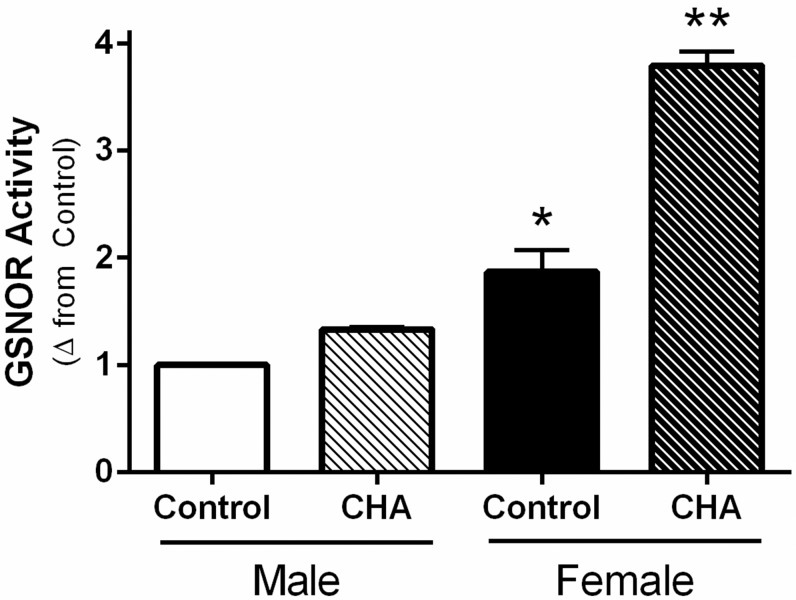
CHA increases GSNOR activity in male and female hearts. GSNOR activity was measured in control and CHA-perfused male and female hearts (male control: clear bar, male CHA: clear hashed bar, female control: black bar, female CHA: black hashed bar; n = 3 hearts/group; *p<0.05 vs. male CHA, **p<0.05 vs. male control, male CHA, female control).

Since we were not able to detect a change in phospho-eNOS with CHA in female hearts, we next examined the effect of CHA on the activity of *S*-nitrosoglutathione reductase (GSNOR) as a potential mechanism underlying the increase in protein SNO levels in female hearts. GSNOR regulates protein SNO levels by mediating catabolism and the genetic deletion of GSNOR results in increased myocardial protein SNO levels [34]. We found that control female hearts exhibited higher GSNOR activity compared to control male hearts, which is consistent with our prior results [25]. Interestingly, perfusion with CHA for five minutes induced a robust and significant increase in GSNOR activity in female hearts, and a more modest increase in GSNOR activity in male hearts (Fig 7). CHA-perfused female hearts exhibited the highest GSNOR activity. In our prior study, we found that male and female WT mouse hearts exhibit comparable GSNOR expression levels [25], indicating that the change in activity is likely independent of GSNOR expression. Since increased GSNOR activity would be expected to favor a decrease in protein SNO levels, GSNOR does not appear to contribute to the CHA-induced increase in protein SNO levels in male or female hearts.

### Post-ischemic reactive oxygen species production is reduced in female hearts

**Fig 8:**
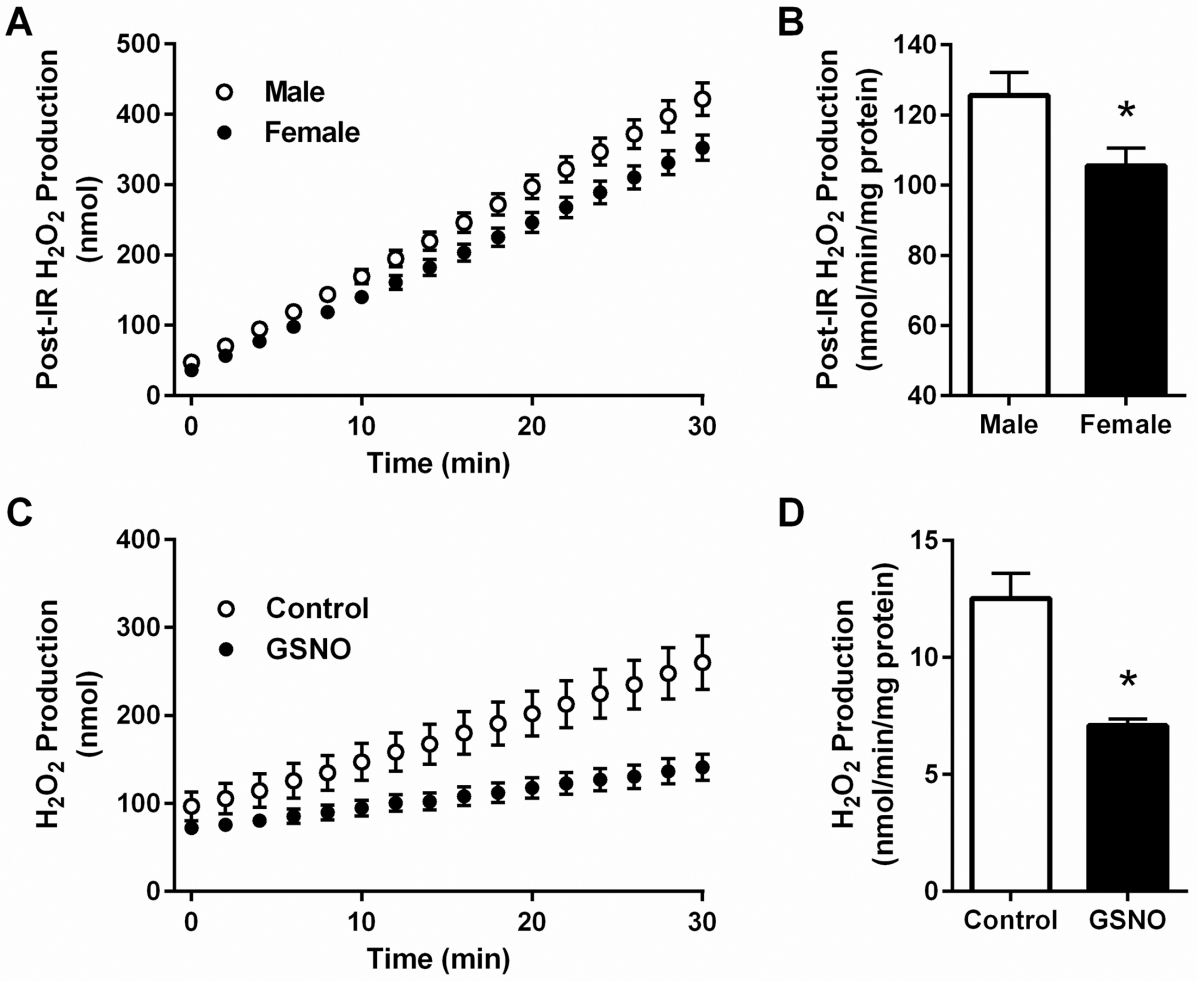
Post-ischemic ROS production is reduced in female hearts. Hydrogen peroxide production was assessed using Amplex Red in post-ischemic heart tissue and with purified α-ketoglutarate dehydrogenase. (A) Hydrogen peroxide production over time (male: open circles, female: closed circles) and (B) the rate of hydrogen peroxide production in post-ischemic male and female hearts (male: clear bar, female: black bar; n = 5 hearts/group; *p<0.05 vs. male). (C) Hydrogen peroxide production over time (control: open circles, GSNO: closed circles) and (D) the rate of hydrogen peroxide production with purified α-ketoglutarate dehydrogenase treated with and without GSNO (control: clear bar, GSNO: black bar; n = 6 replicates/group; *p<0.05 vs. control).

Female hearts consistently showed better post-ischemic functional recovery compared to male hearts, both at baseline and following perfusion with CHA. To investigate a potential mechanism for this enhanced protection in female hearts, we next investigated post-ischemic ROS production in male and female hearts after 20 minutes of ischemia and five minutes of reperfusion using Amplex Red fluorescence as a measure H_2_O_2_ production. Consistent with a previous study in rat heart mitochondria [35], we found that post-ischemic ROS production was significantly reduced in female hearts compared to male hearts (Fig 8a). Interestingly, we found that dihydrolipoyl dehydrogenase was uniquely SNO-modified in the female heart. Dihydrolipoyl dehydrogenase is a component of the alpha-KGDH complex, which is considered to be a major source for the production of ROS [36–38]. Therefore, we next examined the effect of SNO on the ROS production of the alpha-KGDH complex using an *in vitro* assay with purified enzyme. Following a 30 minute incubation with 1 mmol/L GSNO, the ROS production of alpha-KGDH was significantly decreased compared to control (GSNO: -43.4±2.3%), as shown in Fig 8b. As such, the SNO modification of dihydrolipoyl dehydrogenase may contribute to the intrinsic protection observed in the female heart.

## Discussion

In the current study, we demonstrate for the first time that pharmacologic activation of the adenosine A1 receptor enhances SNO protein levels in multiple cellular compartments and induces cardioprotection from I/R injury in male and female hearts, in part via increased phospho-Akt and phospho-eNOS levels. We first used our Langendorff-perfused heart model of I/R injury to demonstrate that pharmacological activation of the adenosine A1 receptor, via CHA perfusion (Fig 1), increases functional recovery in both male and female hearts (Fig 2). These results are consistent with prior work in the male heart [27], but to our knowledge, we are the first demonstrate that adenosine A1 receptor activation can further increase the cardioprotective threshold of the female heart. We next examined Akt phosphorylation at ser473. Consistent with previous reports [33], we observed that baseline Akt phosphorylation was significantly increased in female hearts compared to males (Fig 3a). This likely underlies, in part, the baseline cardioprotective phenotype that we and others have observed in the female heart [16–19, 22–24, 39]. Perfusion with CHA induced a significant increase in Akt phosphorylation at ser473 compared to baseline in both male and female hearts (Fig 3a). eNOS phosphorylation at ser1177 was similarly increased with CHA treatment, but only in male hearts (Fig 3b). CHA did not alter eNOS phosphorylation in female hearts, but eNOS phosphorylation was already increased at baseline in female hearts compared to control males, potentially indicating that there may be some redundancy between adenosine-mediated protection and baseline cardioprotection in females, or that cardioprotective adenosine signaling is different between male and female hearts. It is also possible that the five minute CHA perfusion period was insufficient to resolve a change in eNOS phosphorylation in female hearts. We next utilized SNO-RAC in tandem with mass spectrometry to identify SNO proteins and modification sites in male and female hearts at baseline and with CHA perfusion. Consistent with our previous study [39], SNO protein identifications were more than 50% higher in female hearts at baseline compared to male hearts (Fig 4). Perfusion with CHA induced a modest increase in protein SNO levels in both male (11.4%) and female hearts (12.3%) (Figs 4 and 5), which is consistent with the CHA-induced enhancement of functional recovery observed in our Langendorff-perfused heart experiments (Fig 2). We identified a number of SNO proteins that were unique to CHA-perfused male and female hearts, as well as a number of SNO proteins that were not detected at baseline in either sex, but were identified following CHA perfusion (Table 2). Comparison of the SNO proteins identified with CHA-induced protection to other forms of cardioprotection, namely pre- and post-conditioning, revealed considerable overlap amongst SNO-modified protein targets (Table 3), potentially indicating that SNO may provide cardioprotective effects by targeting a similar protein population, regardless of the form of cardioprotection (i.e., pharmacologic preconditioning, ischemic preconditioning, etc.). For common SNO protein identifications, we used label-free peptide quantification and identified a number of different SNO protein populations (Fig 6). These ranged from SNO proteins that were modified at low levels at baseline in male and female hearts and increased with CHA perfusion only in female hearts, to SNO proteins that were modified at low levels or undetectable at baseline in male hearts and increased with CHA perfusion to levels observed at baseline or with CHA perfusion in female hearts. This latter group of SNO proteins is of particular interest since these proteins may represent important targets in the male heart that when SNO-modified, may induce a cardioprotective phenotype similar to that observed at baseline in the female heart. In addition, we examined GSNOR as an alternative mechanism underlying the CHA-induced increase in protein SNO levels and found that CHA perfusion actually increased GSNOR activity in male and female hearts (Fig 7). Since increased GSNOR activity would tend to favor a decrease in protein SNO levels, this suggests that perhaps GSNOR activity follows changes in protein SNO levels. SNO has been shown to increase GSNOR activity in the lung [40], but we were unable to detect SNO-GSNOR in our proteomic screens. Further, female hearts always performed better following I/R injury compared to male hearts, whether it be at baseline or with CHA perfusion. This can likely be attributed, in part, to the higher level of SNO proteins consistently observed in the female heart and to the modification of specific protein targets like dihydrolipoyl dehydrogenase, which is a member of the alpha-KGDH complex. We showed that treatment of the purified alpha-KGDH enzyme complex with GSNO to induce SNO of the enzyme, reduces the production of ROS (Fig 8b). Consistent with this reduction in ROS production, SNO of alpha-KGDH has been shown to inhibit enzyme activity [41]. We also found that female hearts produced less ROS compared to male hearts after I/R injury (Fig 8a), which may partly explain the enhanced functional recovery that is consistently observed in female hearts. Consistent with these results, mitochondria isolated from female rat hearts after hypoxia-reoxygenation showed less ROS production compared to mitochondria isolated from male hearts [35]. Taken together, these results support a potential mechanism whereby activation of the adenosine A1 receptor leads to enhanced Akt and eNOS phosphorylation, increased SNO protein levels and cardioprotection from I/R injury in both male and female hearts. However, since the effect of CHA on other signaling pathways (i.e., MEK1/2 [42]) was not examined, and we cannot rule out potential contributions from additional signaling pathways.

### Estrogen, nitric oxide and cardioprotection in the female heart

Epidemiological studies show that pre-menopausal women have lower rates of cardiovascular disease compared to age-matched men, but disease incidence increases greatly following menopause [43–45]. This is suggestive of a cardioprotective role for estrogen, but recent hormone replacement therapy trials in postmenopausal women have failed [46, 47]. In animals model of I/R injury (i.e., mouse, rat), female hearts display similar intrinsic protection from injury as we and others have shown [16–19, 22–24, 35, 39, 48]. Studies have also shown that exogenous estrogen protects both male and female hearts from I/R injury in a number of species, including mouse and rabbit [35, 49, 50]. Our group has also shown that selective activation of G-protein coupled estrogen receptor 1 (GPER1), a membrane-bound receptor responsible for the rapid, non-genomic actions of estrogen, induces cardioprotection through the activation of the PI3K and ERK signaling pathways [51]. Our group and others have further shown that female hearts lose sex-dependent cardioprotection following ovariectomy in a number of species, including mice and rats [19, 35, 52]. We have also shown that this protection can be restored in ovariectomized female hearts via administration of 17beta-estradiol (E2) or 2,2-bis(4-hydroxyphenyl)-proprionitrile (DPN) [52]. Interestingly, we also find that E2 or DPN administration increases protein SNO levels in ovariectomized female hearts [52]. GPER1 activation has also been shown to increase eNOS phosphorylation through an Akt-dependent mechanism [53]. These and other studies support a potential role for nitric oxide and protein SNO in the protective effects of estrogen.

In our prior study, we found that female wildtype mouse hearts exhibited higher baseline eNOS expression and phosphorylation, enhanced NO production, and increased protein SNO levels, and associated with this, protection from I/R injury compared to male hearts. We also found that GSNOR activity levels were higher in female hearts compared to males, which would tend to favor lower protein SNO levels. However, female hearts exhibit higher protein SNO levels, as we show in the current study (and in a previous study [25]), suggesting that enhanced GSNOR activity may be necessary to protect against hyper-nitrosylation and the development of nitrosative stress in the female heart. Excessive protein SNO has been shown to contribute to disease pathogenesis with neurodegenerative conditions, neuromuscular atrophy and sepsis [54–56]. In the heart, the effects of many NO donors are also biphasic. For example, we find that administration of 10 μmol/L SNAP, an *S*-nitrosylating agent, induces cardioprotection in the male heart, but this protection is lost when the concentration of SNAP is doubled to 20 μmol/L [57]. Thus, it was unclear whether a further increase in protein SNO in female hearts would be beneficial, as we have shown in the male heart, or detrimental by inducing nitrosative stress. The results of our current study suggest that the ischemic tolerance of the female heart can be further increased with adenosine A1 receptor activation. Female hearts also appear to be able to tolerate a modest increase in protein SNO levels without detrimental effects due to nitrosative stress. Interestingly, GSNOR activity appears to mirror the increase protein SNO levels, perhaps as a protective measure to combat the potential for excessive protein SNO. Future studies will examine the role of sex hormones in the regulation of protein SNO levels and GSNOR activity in the heart, and determine whether a more robust increase in protein SNO is also protective in the female hearts, as is the case for male hearts (i.e., GSNOR^-/-^ heart [58]).

### Common SNO targets in cardioprotection

Protein SNO is a reversible cysteine modification that is stimulus-mediated, spatially localized and targeted to select cysteine residues [59]. Specific mechanisms for the addition and removal of protein SNO have also been identified [34, 60]. As such, protein SNO is a bona fide signaling mechanism in the heart and other organ systems, and does not simply represent a random event. In the context of cardioprotection, we and others consistently find that enhanced myocardial protein SNO levels are generally protective in the setting of I/R injury [1–10, 25]. In the current study, we demonstrate a protective role for enhanced protein SNO levels in a model of pharmacologic preconditioning in male and female hearts. Interestingly, we find that similar populations of proteins are modified via SNO, independent from the model of cardioprotection (i.e., ischemic pre- and post-conditioning, pharmacologic preconditioning, sex-dependent protection) [1–10, 25, 30, 52], and mitochondrial proteins represent some of the most common targets of SNO. For example, we found ANT to be SNO-modified at cys160 in the current study and in a number of previous studies [2, 3, 30]. The VDAC isoforms, namely VDAC1 at cys245 and VDAC2 at cys48, have been identified to be SNO modified in our previous studies [3, 4, 30]. In the current study, not only did we identify modification of VDAC1 and VDAC2 at the same respective modification site, but we also found that VDAC3 was SNO-modified at cys65 with CHA treatment. Interestingly, ANT and VDAC are both thought to potentially play a role in cell death with I/R injury, and both have been reported to play a role in redox sensitive signaling [61, 62]. Additional targets include SERCA2a and many proteins involved in glycolysis. However, specific roles for each of these SNO-modified protein in cardioprotection remains to be determined. Protective roles for many SNO-modified proteins have described in the heart, as we have shown for TRIM72 and CypD [63, 64], but modification of other targets, including XIAP, Drp1, CDK5 and Parkin, have been shown to lead to deleterious effects in the brain [54]. As such, it is possible that SNO of particular protein targets leads to beneficial effects, while the modification of other proteins leads to deleterious effects, but the balance favors reduced injury. Future studies will follow-up on the role of specific SNO-modified protein targets.

### Therapeutic implications

Timely reperfusion is currently the only strategy that consistently reduces infarct size in humans following myocardial ischemia, but adenosine [65–67] and nitrate [68, 69], which can be metabolized to NO, have shown promise in a limited capacity. However, the majority of recent clinical trials of cardioprotective interventions, including those utilizing NO donor compounds, have failed to demonstrate a reduction in infarct size [70, 71] [72–75]. Although our group recently published a study showing that non-failing female human hearts have higher protein SNO levels compared to non-failing male hearts [26], suggesting possible relevance to human physiology, a number of confounding factors may contribute to the loss of protective mechanisms in the clinical setting, including age and/or concurrent pathology. Studies in animal models suggest that cardioprotective signaling is attenuated with aging [76–78], including the loss of adenosine-mediated protection [79]. Many pathological states of the heart similarly abrogate protection. For example, diabetes mellitus has been shown to disrupt cardioprotective signaling, and as such, diabetic hearts cannot be conditioned or cardioprotected [80]. Therefore, age and concurrent pathology has the potential to disrupt the protection afforded by adenosine and protein SNO levels in male and female hearts. Since age and concurrent pathology are critical in terms of translating cardioprotective strategies to the clinical setting and most studies of cardioprotection are performed with young healthy animals, future studies of cardioprotective signaling will need to account for these confounding variables.

## Conclusions

In summary, we have demonstrated that activation of the adenosine A1 receptor increases post-ischemic functional recovery in both male and female hearts. We found that adenosine A1 receptor activation increases phosphorylated Akt (at ser473) and phosphorylated eNOS (at ser1177) levels and enhances the level of SNO proteins in both male and female hearts, likely contributing to the cardioprotective effects of adenosine A1 receptor activation. This study has not only demonstrated the protective effects of adenosine A1 receptor activation in the male and female heart in the setting of I/R injury, but also suggests that changes in protein SNO levels may play a critical role in pharmacologic cardioprotective mechanisms.

## Supporting information

S1 Table SNO protein and peptide identifications from male hearts at baseline as assessed via SNO-RAC in tandem with LC-MS/MS. To view peptide sequences, click on the ‘+’ symbol found on the left side of the spreadsheet; 'Nethylmaleimide' modified cysteine residues are blocked and do not represent sites of SNO. Each of the eight biological replicates are identified in column headings as A2 (Heart 1), B2 (Heart 2), C2 (Heart 3), and D2 (Heart 4), etc. Non-cysteine containing peptides were filtered from the data set (n = 8 hearts/group; FDR: 1%).
(XLSX)

S2 Table SNO protein and peptide identifications from female hearts at baseline as assessed via SNO-RAC in tandem with LC-MS/MS. To view peptide sequences, click on the ‘+’ symbol found on the left side of the spreadsheet; 'Nethylmaleimide' modified cysteine residues are blocked and do not represent sites of SNO. Each of the eight biological replicates are identified in column headings as A2 (Heart 1), B2 (Heart 2), C2 (Heart 3), and D2 (Heart 4), etc. Non-cysteine containing peptides were filtered from the data set (n = 8 hearts/group; FDR: 1%).
(XLSX)

S3 Table SNO protein and peptide identifications from CHA-treated male hearts at baseline as assessed via SNO-RAC in tandem with LC-MS/MS. To view peptide sequences, click on the ‘+’ symbol found on the left side of the spreadsheet; 'Nethylmaleimide' modified cysteine residues are blocked and do not represent sites of SNO. Each of the seven biological replicates are identified in column headings as A2 (Heart 1), B2 (Heart 2), C2 (Heart 3), and D2 (Heart 4), etc. Non-cysteine containing peptides were filtered from the data set (n = 7 hearts/group; FDR: 1%).
(XLSX)

S4 Table SNO protein and peptide identifications from CHA-treated female hearts at baseline as assessed via SNO-RAC in tandem with LC-MS/MS. To view peptide sequences, click on the ‘+’ symbol found on the left side of the spreadsheet; 'Nethylmaleimide' modified cysteine residues are blocked and do not represent sites of SNO. Each of the seven biological replicates are identified in column headings as A2 (Heart 1), B2 (Heart 2), C2 (Heart 3), and D2 (Heart 4), etc. Non-cysteine containing peptides were filtered from the data set (n = 7 hearts/group; FDR: 1%).
(XLSX)
